# Compliance of Healthcare Worker’s toward Tuberculosis Preventive Measures in Workplace: A Systematic Literature Review

**DOI:** 10.3390/ijerph182010864

**Published:** 2021-10-15

**Authors:** Halim Ismail, Naiemy Reffin, Sharifa Ezat Wan Puteh, Mohd Rohaizat Hassan

**Affiliations:** Department of Community Health, Faculty of Medicine, Universiti Kebangsaan Malaysia, Kuala Lumpur 56000, Malaysia; halimismail@ppukm.ukm.edu.my (H.I.); sh_ezat@ppukm.ukm.edu.my (S.E.W.P.); rohaizat@ppukm.ukm.edu.my (M.R.H.)

**Keywords:** compliance, occupational tuberculosis, healthcare workers, tuberculosis prevention measures

## Abstract

Despite several guidelines published by the World Health Organization (WHO) and national authorities, there is a general increase in the number of healthcare workers (HCWs) contracting tuberculosis. This review sought to evaluate the compliance of the HCWs toward tuberculosis preventive measures (TPMs) in their workplace. Both electronic databases and manual searches were conducted to retrieve articles regarding the compliance of HCWs in the workplace published from 2010 onwards. Independent reviewers extracted, reviewed, and analyzed the data using the mixed methods appraisal tool (MMAT) 2018, comprising 15 studies, 1572 HCWs, and 249 health facilities. The results showed there was low compliance toward TPMs in the workplace among HCWs and health facilities from mostly high-burden tuberculosis countries. The failure to comply with control measures against tuberculosis was mainly reported at administrative levels, followed by engineering and personnel protective control measures. In addition, low managerial support and negative attitudes of the HCWs influenced the compliance. Further studies are needed to elucidate how to improve the compliance of HCWs toward the preventive measures against tuberculosis in order to reduce the disease burden among HCWs worldwide.

## 1. Introduction

Tuberculosis is a chronic communicable infectious disease with a long history. Tuberculosis is a major cause of ill health and among the top 10 causes of death globally [1]. It is an airborne infectious disease that spreads easily among humans and a common nosocomial infection that widely affects healthcare workers (HCWs). Tuberculosis is considered a serious problem among HCWs. It has been recognized as an occupational hazard since the 1950s when many hospitals succumbed to an outbreak of tuberculosis among patients and health staff [2]. However, it was only recently that this problem was highlighted. In fact, most countries only started to report the incidence of tuberculosis among HCWs after observing the annual increase in the number of cases. The problem was greater in a country with a high burden of tuberculosis disease in the community. Tuberculosis burden worldwide was affected by the COVID-19 pandemic, which caused diagnostic delay and disruption of tuberculosis care services, causing increase in clinical severity [3]. The World Health Organization (WHO) revealed that the incidence of tuberculosis among HCWs is increasing yearly, and in most countries, it exceeds the community incidence of the disease. In 2019, a total of 22,314 tuberculosis cases among HCWs were reported to the WHO by 76 countries, with India contributing the highest numbers by accounting for 47% of the total cases, followed by China with 18% [1]. Based on the reports to the WHO, the notification rate of tuberculosis among HCWs was more than two times the rate in the general adult population in 42 countries [1,4]. Several studies also reported the prevalence and incidence rate of contracting tuberculosis disease were much higher among HCWs compared to the community [5,6,7,8,9,10]. WHO also reported that latent tuberculosis infection (LTBI) and tuberculosis disease among HCWs were 5.7 and 2–10 times greater than the estimates in the general population in low and high-income countries, respectively [11,12,13]. In addition, the risk for hospitalization with multi-drug resistance tuberculosis (MDR TB) is approximately 5–6 times higher among HCWs than in the general population [14]. More importantly, tuberculosis disease has been reported to be associated with depression [15,16], which highlights the likelihood of higher severity when HCWs contract the disease. 

The WHO have published several guidelines on infectious diseases prevention controls in the workplace in order to reduce the risk of HCWs contracting nosocomial infection diseases in health facilities. Given the fact that HCWs are considered as a high-risk group in contracting tuberculosis based on their occupation, a specific guideline in preventing and reducing the risk of contracting occupational tuberculosis disease among HCWs was produced worldwide [17,18,19]. Additionally, many countries have published guidelines locally to confront these similar issues. The tuberculosis prevention and control guideline for HCWs published by WHO consists of three main strategies: 1. administrative or managerial control measures, which includes tuberculosis infection control (TBIC) plan and tuberculosis risk assessment and HCWs training; 2. environmental control measures, such as proper ventilation to remove contaminated air; and 3. respiratory protective devices or personnel protective equipment measures, including protective surgical mask for suspected tuberculosis patients and proper respirator devices for HCWs [4]. Administrative control was the first line in the hierarchy of defense as strongly recommended by the WHO, followed by environmental control and respiratory protective measures. 

Administrative control aims to prevent HCWs and patients from being exposed to tuberculosis and reduce the transmission by ensuring rapid diagnosis and treatment of presumptive tuberculosis patients. Environmental control aims to prevent the spread of tuberculosis by reducing the concentration of infectious respiratory aerosols in the air. Environmental control should be in place together with administrative controls, as it requires budgeting and financial supply for the measures to be appropriately implemented. On the other hand, respiratory protective measures aim to double protect HCWs individually, especially those working in a high-risk workplace [20]. By rule, as a healthcare worker, the risk of a healthcare worker contracting tuberculosis from the workplace is supposed to be lower than in the general population due to healthy worker effects. Nevertheless, the reverse is the case as HCWs remain the most affected with the highest burden of the disease worldwide. 

Notwithstanding the several guidelines published in different countries to either regulate the activities or control measures required to reduce tuberculosis burden among HCWs, most of these guidelines focus on the policies, management, and infrastructure. None of these guidelines include the behavioral parts of the HCWs to create a complete and effective template to minimize their exposure to the disease. Adherence and compliance to tuberculosis prevention guidelines in the workplace are very important and crucial to reduce the burden of tuberculosis disease among HCWs. Furthermore, compliance of HCWs toward the control measures in the guidelines may contribute to the success of the preventive measures in reducing the burden of tuberculosis among HCWs. In addition to other issues such as financial constraints and high managerial involvement, weak implementation with less monitoring and no compliance to the guideline were the main contributors toward increasing incidence of tuberculosis among HCWs in most countries [10,21,22]. 

There is an existing review that analyzed the effectiveness and the need for implementation of TPMs according to the guideline provided by the WHO [23]; however, no recent literature review has been published regarding the compliance of HCWs toward TPMs in the workplace. Considering the importance of compliance of HCWs in implementing and following the guideline properly to reduce the risk of transmission of tuberculosis disease, this review evaluates the compliance of HCWs toward TPMs in the workplace to reduce the incidence of tuberculosis among HCWs due to occupational risk. 

## 2. Materials and Methods

### Search Strategy

A systematic literature search strategy was employed in this review to retrieve complete and comprehensive articles related to the research topic. A detailed protocol was used for the systematic review, whereby all the relevant studies obtained from the literature search were extracted and examined. This procedure step was run systematically in seven steps as follows: develop a research question, select suitable keywords in relation to the topics, define the databases that are relevant for the search process, determine the limitations of the search (i.e., a timeframe of the articles), the languages used and the nature of the documents, developed a review strategy, screening and examine the desired literature, and analyzing the selected articles.

The research question in this study was formulated using the Population, Intervention, Control, and Outcomes (PICO) strategy. PICO is a tool that assists an author to developed suitable research questions for a review [24,25]. In this review, since there was no intervention, exposure was used as the most suitable and alternative concept. Based on these concepts, three main aspects were included in the review, as shown in Table 1.

In order to select the number of articles relevant to the research topic, the identification of keywords was conducted, which aimed to provide more options and selection of databases to search for more related studies. All similar terms to the keywords were identified using an online Thesaurus dictionary and through past research articles. The main databases comprising Scopus, Web of Sciences (WOS), and PubMed were first searched for articles relevant to the topic. Thereafter, articles were manually selected using other databases such as Google Scholar and manually picked or hand-picking techniques. The search process was executed using advanced searching techniques such as Boolean operators, phrase searching, truncation, wild cards, and separate field code functions, followed by combining these searching techniques into a full searching string, as shown in Table 2.

All the identified articles were sorted, and duplicates were removed. Subsequently, the articles were screened for eligibility according to the inclusion and exclusion criteria. For studies to be included in the review, the articles must have been published between 2010 and 2020 and focused on TPMs in the workplace. The relevant articles published in this timeframe were selected as TPMs in the workplace were changed rapidly following the latest technology with rapid progression on new drugs, new interventions, and new diagnostic tools. The other inclusion and exclusion criteria are listed in Table 3. 

All selected articles fulfilling the inclusion and exclusion criteria were then independently and manually read and analyzed by the authors. Specifically, the title and abstracts and were discussed to ensure their eligibility in the review for the objective to be achieved. All studies were considered and included in the analysis, irrespective of whether qualitative or quantitative methods were employed in the articles. The electronic database search was conducted from October 2020 to April 2021, and the review was conducted in accordance with the Preferred Reporting Items for Systematic Reviews and Meta-Analyses (PRISMA) [26].

For the assessment of the risk of bias and quality evidence, the authors verified whether the relevant articles met the objective and inclusion criteria. All the articles were individually analyzed based on the objective of the study, and quality appraisal was performed using mixed method quality appraisal (MMAT) 2018 [27]. These tools were selected for the appraisal stage in view of their capacities to appraise quantitative, qualitative, and mixed-method study designs. Furthermore, these tools are useful for the appraisal of the quality of empirical studies, including primary research based on observation and stimulation. In this study, peer reviews were conducted for all the selected articles to avoid cases of dispute, and all authors agreed that each selected article must attain a minimum of moderate level quality to be included in the analysis. The summary of quality appraisal grading is shown in Table 4. 

## 3. Results

### 3.1. Descriptive Analysis

**Figure 1:**
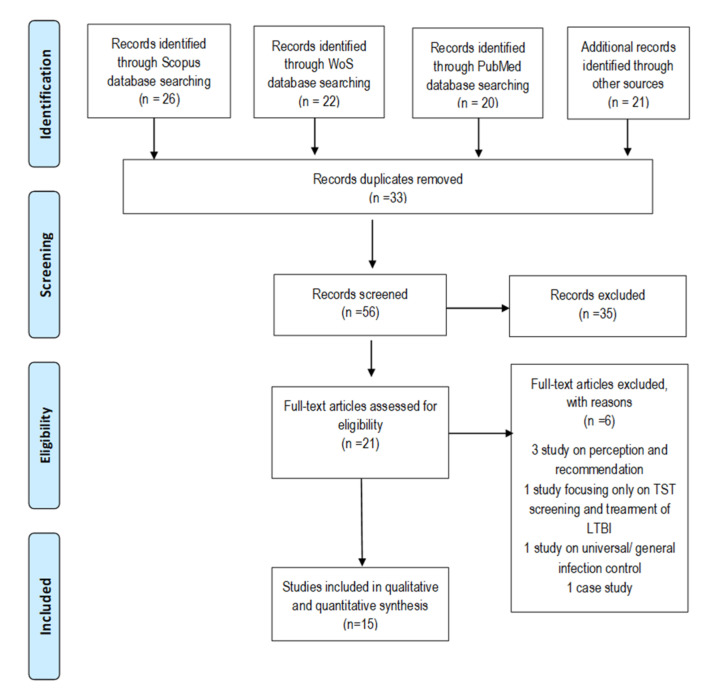
PRISMA flow diagram.

A total of 88 articles were retrieved from the literature search. Upon data sorting, 33 duplicated articles were removed from the initial analysis, while the remaining 55 articles were eligible for further assessment. All articles were then screened by assessing their abstracts to determine if they fulfilled the study objectives. A total of 21 articles were found to be eligible to be included in the final and detailed analysis. From the 21 articles, six were excluded due to one or more of the following reasons: 1. the study only assessed the HCWs’ perception regarding their compliance toward TPMs; 2. focused on one or two selected control measures, such as screening of HCWs using tuberculin skin test (TST) and treatment of latent tuberculosis infection (LTBI); and 3. the study only described the situational status of HCWs’ adherence toward TPMs using a case scenario. Figure 1 shows the results of the search strategy and study selection process according to the PRISMA guidelines. The six excluded articles are listed in Table 5 with the reason for exclusion and the relevant findings [20,28,29,30,31,32]. 

In this review, excluding one study [33], the remaining 14 studies were carried out in countries with a high burden of tuberculosis disease [34,35,36,37,38,39,40,41,42,43,44,45,46,47]. Eight studies were conducted in the South Africa state. All the studies were conducted between 2011 and 2020 based on the WHO guidelines on the prevention of tuberculosis disease among HCWs or in a segregated setting that was published earlier in 2009 [4] and their respective local guidelines. This review evaluates HCWs’ compliance to TPMs based on the measurement levels, namely, administrative control measures, engineering control measures, and personal or respiratory protective equipment specified in the usage of respirator N95 among HCWs. Based on the final analysis, 11 of the studies used mixed-method study design by combining quantitative and qualitative methods, especially via observational study design or site auditing at the facility. Various ranges of HCWs types were selected according to the study’s objective, with the majority being nurses, doctors, and HCWs in the clinical field [33,34,35,36,39,40,43,44,45]. Two studies employed facilities as their respondent or study subjects [37,41]. The majority of respondents were females ranging from 44% to 90.2% of the total participants in each study. Furthermore, the respondents’ mean age ranged from 25.8 to 45 years old. A summary of the descriptive findings of the articles is presented in Table 6.

The majority of the studies (80%) concluded that the compliance of HCWs toward TPMs was poor, whereas three studies inferred that the HCWs’ compliance was good [36,37,45]. One of the studies stated that HCWs’ had a good perception of complying with TPMs, but it was poorly implemented based on the researcher’s assessment. Studies conducted using mixed-method design showed some discrepancies result between an interview or self-administered questionnaire and the observational methods. Table 7 shows the summary of the findings on HCWs’ compliance relative to the level of control measures. 

This review summarized three main control measures: 1. administrative and managerial control measures focusing on present tuberculosis guidelines or policies in the workplace, present committee or person in charge of TPMs in the workplace, HCWs’ training and surveillance measures, tuberculosis education activities, and any other relevant control measures pertaining to management decision or plan; 2. engineering control measures; the factors regarding ventilation system in the workplace that is suitable and manage to halt or reduce the contaminated air with tuberculosis bacteria and additional equipment, such as ultraviolet germicidal irradiation (UVGI); and 3. the last preventive strategy is personal protective measures or respiratory protective measures, which are mainly based on the availability and usage of respirators provided to HCWs and conducting a fit test before use. 

#### 3.1.1. Administrative and Managerial Control Measures

All the studies included in the final analysis evaluated the TPM focused more on administrative levels, as it is the most important level of control measures in preventing tuberculosis infection in all areas. Basically, administrative measures comprise all plans and activities executed in order to ensure that the existing TPMs are properly adhered to by HCWs. All the eligible studies employed different assessment methods, either based on the guideline provided by the WHO or the local authority. From the 13 studies that evaluated existing TBIC guidelines at the workplace, only seven of them [36,37,39,43,44,46] reported that more than 50% of the respondents were aware of the guidelines (i.e., WHO or national guideline). Additionally, only seven studies [35,38,39,40,41,47] considered respondents’ present awareness about the committee or person responsible for handling and managing TPMs in their facilities. 

Further analysis revealed that four studies [36,39,44,47] reported that more than 50% of HCWs were trained for tuberculosis prevention guidelines in workplaces, whereas two studies [33,34] highlighted that surveyed respondents were not trained for tuberculosis prevention. Of the six studies evaluating tuberculosis surveillance and screening measures for HCWs, only four involved the screening of more than 50% of the HCWs [33,34,37,39,44,45]. In terms of educating patients about tuberculosis, seven out of the nine studies that considered the aspect involved more than 50% of the respondents. Specifically, patients were educated on general aspects of tuberculosis disease or cough hygiene etiquette in their workplace [36,38,40,42,44,45,47]. 

#### 3.1.2. Engineering Control Measures

A total of 12 studies assessed engineering control relating to ventilation in workplace facilities; seven of them [34,36,37,38,42,44,47] reported either an overall satisfactory adherence or more than 50% of HCWs affirming that the ventilation system in their workplace was good. However, six studies found that the implementation of additional air cleaners such as UVGI was either absent or minimal present in their workplace.

#### 3.1.3. Personnel Protective Equipment (PPE) Control Measures 

A total of 14 studies assessed activities relating to PPE measures. More than 50% of HCWs surveyed in eight studies stated that their workplace had PPE available, but only three studies reported that more than 50% of respondents practiced good PPE usage [34,36,42]. Likewise, only six studies evaluated the provision of fit-testing activities to HCWs, and only two studies reported that more than 50% of the respondent underwent fit testing [45,46]. Mixed-method study designs were used in four studies, and there were disparities between the respondents’ answers via interview or questionnaire and observational components organized by the researcher. Specifically, most of the studies reported low compliance to the activities by observation as compared to the self-reports by respondents [35,36,38,42]. 

## 4. Discussion

The overall findings from the reviewed studies revealed poor compliance or inadequate implementation of TPMs in the workplace. This may contribute to the continuous increment of the prevalence of tuberculosis among HCWs worldwide [30,48,49]. The guideline for TPMs among HCWs was published early in 1999 by the WHO and updated from time to time according to prevailing situations. Most of the countries, especially those with a high burden of tuberculosis, should have effective implementation of TPMs since it is part of the general prevention and control of tuberculosis programs. 

The first step in the prevention and control hierarchy as per guided by WHO is administrative control measures. It is recommended that all policymakers should implement various strategies in administrative control measures, especially in countries with high tuberculosis burden. For TPMs at the administrative level to be successful, strong commitments from the higher level of organization and political will and effective leadership are important. A basic requirement of the implementation, such as a guideline or policy about the implementation of control measures in the workplace with the involvement of all HCWs, should be emphasized and well distributed to the knowledge of all the HCWs. This is because all the subsequent strategies and planning activities will follow the recommendations in the guideline. 

Several studies reported the importance and the need for effective administrative control measures to reduce not only the risk of tuberculosis transmission but other hospital-acquired infections in the workplace [50]. This review showed that there is a poor establishment of the guideline in countries with regards to the availability or the accessibility to HCWs at the ground levels. Most of the studies also highlighted the need to establish the policy or the guideline to ensure HCWs’ adherence to other measures [22,51]. In addition, there are conflicting issues in the present guideline that hinder HCWs from applying proper preventive measures [30]. This problem gives rise to false information and a poor understanding of the disease and control measures that should be adhered to by the HCWs. As noted by Gilson, it is the frontline workers who ultimately translate policy intentions into practices [52].

The establishment of a committee in controlling the transmission of infection in the workplace, including tuberculosis, was important to ensure that the proposed measures were properly implemented. A study by Godrey (2016) showed that the establishment of a committee was significant to oversee the compliance of HCWs to disease preventive measures. Specifically, the presence of such a committee enhanced the results of HCWs’ compliance compared to facilities where no committee manages the affairs [37]. The presence of the committee assists the HCWs in managing and implementing the control measures besides being an observer for their activities. On the other hand, the observer or auditing person plays the role of reminding the HCWs to implement proper control measures during their daily routine. This was also highlighted as a socially desirable effect [42], whereby HCWs knowing that their activities are being observed will perform well in adhering to all the guidelines. Nevertheless, contradicting results were reported by Naidoo (2012) as there was no significant difference in the adherence to preventive measures in clinics with and without infection control committees [41]. The study employed an observation method, which might have induced a bias since the staff was aware that they would be observed or audited during their daily routine and practices, otherwise known as the Hawthorne effect [53]. 

Training of HCWs is very important and should be carried out persistently in order to continuously update them about the latest and current policies. In this review, training was noted to be provided minimally to HCWs. A similar finding was also reported in one study as the training of HCWs was not considered important [31]. However, two studies addressed the relevance of training, where HCWs were constantly trained to improve their practices of TPMs [22,28]. In fact, training was identified as one of the barriers to implementing the control measures. By conducting proper training relating to TPMs, including the usage of PPE protection, it is expected that the knowledge of HCWs will improve. Many HCWs are aware of their risk of contracting tuberculosis disease from workplaces. Generally, they also had good knowledge regarding the transmission of the disease, but it was not put into action. This knowledge–action gap was highlighted in several studies included in this review [5,28,33,46,54].

From this review, only one study evaluated the issue of budgeting or financial allocation to tuberculosis prevention programs [34]. Financial allocation and sufficient budgeting are vital to ensure the sustainability of TPMs. Issues on availability and consistent supply of PPE, additional devices in controlling contaminated air, structural or facility modification to implement the proper prevention measure were cost demanding. Allocation of staff or increased manpower to assist in establishing TPMs also requires a lot of financial allocation. It is important to evaluate the associated cost of the risk of tuberculosis transmission. Joshi (2006) concluded that the lack of resources in developing countries makes it difficult for hospitals, clinics, and healthcare centers to implement adequate TPMs [6]. Lack of funding arises from the management’s little priority for TPMs and the fact that services provided for free did not generate sufficient revenue [47]. 

There is also the need to strengthen the surveillance system of tuberculosis among HCWs. This will assist in protecting the HCWs by early identification of occupational diseases, initiation of prompt treatment, and necessary interventions among the staff and facilities. Surveillance systems for HCWs regarding TPMs are executed using various available methods in the facilities, such as tuberculin skin test (TST), chest X-ray, or even IGRA. It is pertinent to comply with the screening activities, but it is less highlighted. This review reported low compliance of HCWs in the surveillance system, either by individual or organization. This finding corroborates the reports by Joseph (2004) [39], whereas Flick (2017) highlighted the rising concern over the state of routine symptoms screening for tuberculosis, as most HCWs did not agree with the procedure and complying with it [48]. The risk of contracting active tuberculosis disease among HCWs was also being highlighted among those with LTBI in Germany [55]. 

Education on tuberculosis should be delivered in all health facilities with or without the presence of tuberculosis patients since HCWs were exposed to the patients who hail from unknown health backgrounds. This review showed that various factors contributed to the minimal implementation of delivering tuberculosis education in the workplace. Tuberculosis education was noted to be provided most to patients compared to HCWs. Although the HCWs were expected to be knowledgeable about tuberculosis, their overall practice in relation to TPMs was still poor. Most of the HCWs in the interview claimed to adhere to TPMs, but it was not reflected following the researcher’s observation at the facility level. Disseminating the guideline and tuberculosis prevention plan alone to HCWs will not change them in practicing proper TPMs. They need to be educated to improve their understanding of the importance of practicing the control measures and the possible consequences of doing the opposite [30,54,56,57]. 

Engineering control measures are the second level of TPMs that should be strengthened, especially in health facilities where there is a higher risk of cross-infection between patients and susceptible HCWs. Although outdoor transmission of tuberculosis is possible, indoor transmissions are more efficient due to limited air dilution and the proximity of occupants. Among environmental controls, natural ventilation and UVGI air disinfection are the most cost-effective choices in reducing the transmission of contaminated air with tuberculosis bacteria in the workplace [58]. This review revealed that most facilities were using mixed and natural ventilation, but the usage was not optimized. For instance, the windows and doors remain closed when they are consulting tuberculosis patients. Despite the natural ventilation system in the facilities, such practices increase the risk of contracting tuberculosis disease. Furthermore, additional air cleaner such as UVGI was present in a few facilities, but their maintenance was questionable. Other barriers identified in the implementation of good engineering control measures were the structure of the facilities themselves, as most were not compatible with health services usage. This was complicated by the increasing number of patients attending the facilities per session, leading to overcrowding and congestive. Difficulties in separating presumed tuberculosis patients or those with cough symptoms from other patients due to lack of space or facility design were identified by most HCWs in the various reviewed studies [30]. 

In facilities with ineffective control measures, specifically managerial measures and incomplete or neglected environmental measures, the responsibility to protect the HCWs lies on the individual. PPE measures must be fully established and made available for HCWs at all times. Nevertheless, this review showed that most HCWs were less informed about PPE prevention measures, not adequately trained on proper PPE usage, and most of them did not undergo proper fit-testing to ensure they were fully protected. According to Jones (2017), an increment of about 95% of compliance to respiratory disease preventive measures would eliminate about one-third of pulmonary tuberculosis infections [59]. However, this aspect of control is the last level of defenses after the managerial and engineering controls. This finding is consistent with the reports from one study, as HCWs were not knowledgeable in using PPE, which led to false or improper usage [30].

## 5. Limitation and Suggestion

The findings of this review were only confined to a selected search engine that had been chosen by the authors given the most common articles related to the research topic. The possibility of missing other papers that were not included in the selected database cannot be ruled out. Therefore, the searching process was complemented using a manual search method. Likewise, studies written in languages other than English were not included in this study, which might be a source of selection bias. Other limitations included the fact that qualitative studies are mostly designed to assess HCWs’ perception toward compliance to specific activities executed in their workplace, thereby reducing the chances of generalizing the results. However, the strengths of this study are well-acknowledged. In addition to employing the PRISMA guidelines in the study selection process and reporting, articles involving the use of mixed-method design (qualitative and quantitative) were included in this review. This was performed to generate results as generalized as possible. Another limitation is that this review may be limited to African countries as 13 studies selected were conducted in Africa within a developing country with a high burden of tuberculosis disease. Thus, it may not represent other developed countries or other developing countries from other regions. 

Whenever possible, a self-admitted study design should be accompanied by direct observation to retrieve robust data. The results of this review could be employed as a guide to improving the current implementation of the national TPMs, especially regarding the prevention of occupational tuberculosis. In addition, to improve the existing guidelines or policies, it should be followed by strategies to change HCWs’ attitudes and behavior to strengthen the prevention measures. The other co-findings in the reviewed articles, such as economic burden either at national levels or specific health facilities, human resources, and individual policies, were rarely explored. This knowledge gap should be considered in future research. 

## 6. Conclusions

This review revealed low compliance of HCWs toward TPMs in the workplace, especially in countries with a high burden of tuberculosis disease. A total of 15 studies with 1,572 HCWs and 249 health facilities, mostly from countries with high tuberculosis burden, reported low compliance of HCWs toward TPMs in their workplace. The administrative level of control measures was identified as the main aspect where HCWs showed a high degree of poor compliance, followed by engineering and personnel protective control measures. Most studies reported that low managerial support is an important factor influencing HCWs’ compliance besides their negative attitudes. Factors affecting the implementation of necessary control measures in health facilities included lack of funding and financial support. Urgent plans and improving existing guidelines regarding TPMs are required in order to face the challenges and reduce the burden of tuberculosis among HCWs in the workplace. Ignoring these recommendations may impair the sustainability of the health system as a whole. 

To the best of our knowledge, this is the first attempt to perform a systematic review evaluating the compliance of HCWs toward TPMs in workplaces with the aim to identify the gap in the implementation of the measures. This review may provide guidance and be an eye-opener to policymakers in countries struggling with the tuberculosis burden among HCWs. 

## Figures and Tables

**Table 1:** PICO strategy in developing research question.

**Population/Person**	**HCWs OR Healthcare Providers**
Exposure	Tuberculosis prevention measures or activities in the workplace
Outcome	Compliance of the health care workers

**Table 2:** Full search string used in selected databases (Scopus, Web of Science, and PubMed).

Topic	Scopus	Web of Science	PubMed
Compliance of healthcare workers toward tuberculosis prevention measures	(“healthcare worker *” OR “health person *” OR “health worker *” OR “doctor *” OR “nurse *” OR “health provider” OR “healthcare provider *”) AND (“tuberculosis prevention measures *” OR “tuberculosis infection activities *” OR “tuberculosis infection control *” OR “TB infection control *” OR “occupational tuberculosis” OR “occupational TB”) AND (compliance * OR adhere * OR obedience *)	(compliance * OR adhere * OR obedient *) AND (“tuberculosis prevention measures *” OR “tuberculosis infection activities *” OR “tuberculosis infection control *” OR “TB infection control *” OR “occupational tuberculosis” OR “occupational TB”) AND (“healthcare worker *” OR “health person *” OR “health worker *” OR “doctor *” OR “nurse *” OR “health provider” OR “healthcare provider *”)	(“healthcare worker *” OR “health person *” OR “health worker *” OR “doctor *” OR “nurse *” OR “health provider” OR “healthcare provider *”) AND (compliance * OR adhere* OR obedience *) AND (“tuberculosis prevention measures *” OR “tuberculosis infection control *” OR “TB infection control *” OR “occupational tuberculosis” OR “occupational TB”)

*—symbols in table two refer to a search technique that we used in searching comprehensive keywords for this review. Another useful search technique is called truncation-a feature which allows you to replace one or more letters in a word with a symbol, usually an asterisk (e.g., farm *). Truncation broadens your search, allowing you to easily search for many words at once.

**Table 3:** The criteria for articles selection.

Criteria	Inclusion	Exclusion
Timeline	2010–2020	Before 2000
Publication type	Article journal, original paper, conference paper	Editorial paper, narrative paper, newspaper, and review paper
Language	English, Malay	Non-English or Malay

**Table 4:** Summary of quality appraisal grading.

First Author (Year)	Screening Questions		1. Qualitative				2. Quantitative RCT					3. Quantitative Nonrandomized					4. Quantitative Descriptive			5. Mixed Methods				Final Quality Grading				
	S1	S2	1.1	1.2	1.3	1.4	1.5	2.1	2.2	2.3	2.4	2.5	3.1	3.2	3.3	3.4	3.5	4.1	4.2	4.3	4.4	4.5	5.1	5.2	5.3	5.4	5.5	
Chapman (2017) [33]	Y	Y	Y	N	Y	Y	Y																					M
Nazneen (2021) [34]	Y	Y																					Y	Y	Y	Y	Y	H
Engelbrecht (2018) [35]	Y	Y																					Y	Y	Y	Y	Y	H
Engelbrecht (2016) [36]	Y	Y																					Y	Y	Y	Y	Y	H
Godfrey (2016) [37]	Y	Y	Y	Y	Y	Y	Y																					H
Kuyinu (2016) [38]	Y	Y																					Y	Y	Y	Y	Y	H
Malangu and Mngomezulu (2015) [39]	Y	Y																					Y	Y	Y	Y	Y	H
Mugomeri (2015) [40]	Y	Y											Y	Y	Y	Y	Y											H
Naidoo (2012) [41]	Y	Y																					Y	Y	Y	Y	Y	H
Kanjee (2011) [42]	Y	Y																					Y	Y	Y	Y	Y	H
Malotle (2017) [43]	Y	Y	Y	Y	Y	Y	Y																					H
Tamir (2016) [44]	Y	Y																					Y	Y	Y	Y	Y	H
Nawa (2020) [45]	Y	Y																					Y	Y	Y	Y	Y	H
Mekebeb (2019) [46]	Y	Y																					Y	Y	Y	Y	Y	H
Kuyinu (2019) [47]	Y	Y																					Y	Y	Y	Y	Y	H

Y—yes; N—no; C—can’t tell; M—moderate; H—high.

**Table 5:** Studies excluded and the reasons for exclusion.

Reference	Title of Articles	Reason for Exclusion	Relevant Findings
Chapman 2018 [28]	Health care workers’ recommendation for strengthening TBIC in the Dominican Republic	This qualitative study described the HCWs’ recommendation for strengthening adherence to TB infection practices in their health institutions across the Dominican Republic.	Six emerging themes were identified by the researchers. The important information to be considered to improve HCWs’ adherence in practicing TPMs in their workplace were: (1) The importance of education and training regarding tuberculosis diseases and their preventive measures; (2) Administrative policies are the most important factors in the implementation and planning of TB infection control measures at all levels; (3) Strengthen the infrastructure policy; (4) Improved economic allocations for TPMs and research.
Joseph 2004 [29]	Factors influencing health care workers’ adherence to work site tuberculosis screening and treatment policies	This exploratory qualitative study identified factors influencing the HCWs’ perception toward adherence to the policy of tuberculin skin test (TST) in identified nosocomial tuberculosis and policy of LTBI treatment. This study focused on TST and LTBI treatment only.	Lack of TB knowledge, especially in its transmission and tuberculosis disease among HCWs, was a consistent theme identified throughout the study.
Zinatsa 2018 [30]	Voice from the frontline: barriers and strategies to improve tuberculosis infection control in primary health care facilities in South Africa	This qualitative study explored and identified factors that influence TBIC behavior at primary health clinics within a high tuberculosis burden district and elicited the recommendation for their HCWs for improvement of TBIC in their primary health clinic.	Major barrier elicited in improving the adherence of the HCWs toward tuberculosis prevention measures in view of the HCWs perception was: (1) Poor training for HCWs, especially on the tuberculosis prevention guideline; (2) The presence of conflicting guidelines in some of the clinics; (3) Low level of motivation among HCWs itself; (4) The feelings of powerlessness among the HCWs; (5) Negative attitudes of HCWs; (6) Poor district health support.
Engelbrecht 2015 [31]	Tuberculosis and blood-borne infectious disease: workplace condition and practices of healthcare workers at three public hospitals in the Free State.	This study assessed workplace conditions and practices regarding air- and blood-borne infections in a public hospital in the Free State in which specifically on tuberculosis dan hepatitis transmission. The study did not assess the compliance of the HCWs following three main activities under TPMs; it only focused on the screening of tuberculosis among HCWs and usage of proper respirator activities.	(1) Physicians were less compliant with hand hygiene and were associated with lower rates of tuberculosis screening. (2) Workplace audits highlighted infection control hazards, including improper use of N95, a lack of available soap, and inadequate availability of sharp containers. (3) Lack of training contributes to the low adherence of the HCWs, in which half of the respondents were found not wearing a respirator when needed as a simple basic precaution to themselves.
Verkuijl 2016 [20]	Protecting our front-liners: occupational tuberculosis prevention through infection control strategies	This study focused more on narrative or write-up study on four levels of TPMs suggested by WHO and the case study of the implementation situational analysis in sub-Saharan African countries.	(1) Poor implementation of TBIC activities in low- and middle-income countries is a substantial challenge affecting efforts to reduce tuberculosis transmission to HCWs. (2) Poor facility infrastructure, building design, and inclement climate often result in poor natural ventilation. Overcrowding, lack of space, and lack of outdoor waiting areas are further challenges for effective TBIC. (3) Stigma is a real challenge in ensuring the adherence of HCWs toward all the prevention measures.
Adu 2020 [32]	Perceived health systems barriers to tuberculosis control among health care workers in South Africa	This study was about the perception of the health care workers on the health system barriers to prevent tuberculosis transmission among HCWs. This study also documented the shortcomings in the implementation of clinical practices guidelines in healthcare and typically drew attention to personal, guideline-related factors.	Deficiencies in the implementation of recommended infection control and TPMs are unlikely to be corrected until health system barriers are addressed. Health system barriers were identified: (1) Leadership and government were top-down and fragmented; (2) Lack of funding was a major barrier; (3) Insufficient staff trained in TBIC; (4) Occupational health services were not comprehensively available; (5) Ability to sustained protective technologies was questioned.

**Table 6:** Descriptive analysis of the studies.

First Author, Year. Study Site	Burden of TB in the Country	Study Type	Sample Size/Type of HCW	Demographics of Respondents	Administrative	Engineering	PPE	Knowledge	Compliance’s Outcome
Nazneen 2021. Bangladesh [34]	High	Mixed method	59 HCWs/11 health settings 28 physicians 11 nurses 19 laboratory workers 1 project director	66% female Mean age: 45 years Mean service: 10 years	Poor	Good	Poor	Poor	Poor
Engelbrecht 2015. South Africa [31]	High	Mixed method	41 PHC 41 TB nurses	90.2% female Mean age: 49.9 years Mean service: 5.95 years	Poor	Poor	Poor	Poor	Poor
Chapman 2017. Dominic Republic [33]	Intermediate	Qualitative	9 HCWs 7 physicians 2 nurses	44% female	Poor	NA	Poor	Good	Poor
Engelbrecht 2016. South Africa [36]	High	Mixed method	236 HCWs/41 facilities 202 nurses 34 community health workers	87.7% female Mean age: 44.2 years Mean service: 6.4 years	Good	Good	Good	Good	Poor
Godfrey 2016. Low middle-income country [37]	High	Qualitative	33 clinical research sites funded by NIAID	-	Good	Good	Good	NA	Good
Kuyinu 2016. Nigeria [38]	High	Mixed method	20 facilities 10 HCWs	NA	Poor	Good	Poor	Good	Poor
Malangu 2015. South Africa [39]	High	Mixed method	52 facilities 89.1% nurses	89.1% female Mean age: 44.7 years	Good	Poor	Good	Good	Poor
Mugomeri 2015. South Africa [40]	High	Quantitative	55 nurses 2 hospitals	76.3% female Mean age: 35 years Mean service: 9 years	Poor	Poor	Poor	NA	Poor
Naidoo 2012. South Africa [41]	High	Mixed method	51 PHC	-	Poor	Poor	Poor	NA	Poor
Kanjee 2011. South Africa [42]	High	Mixed method	57 HCWs 43.8% professional/enrolled nurse	75.4% female Mean service: 5 years	Poor	Poor	Good	Good	Poor
Malotle 2017. South Africa [43]	High	Qualitative	285 HCWs 50.7% nurses 5.3% doctors 28.9% support	73.3% female Mean age: 41.4 years	Poor	NA	Poor	NA	Poor
Tamir 2016. Northwest Ethiopia [44]	High	Mixed method	647 HCWs/ 15 PHC 53.1% nurses 12.8% health off 11.1% lab 10.9% midwives 12% pharmacist	61% male Mean age: 25.8 years	Poor	Poor	Poor	Good	Poor
Nawa 2020. Namibian [45]	High	Mixed method	3 hospitals/ 171 HCWs 27% doctors 20% nurses 16% env health practitioners	72% female	Good	Poor	Good	Good	Good
Mekebeb 2019. South Africa [46]	High	Mixed method	2 hospitals/ 2 occupational health and infection control persons	NA	Poor	Poor	Good	Good	Poor
Kuyinu 2019. Nigeria [47]	Yes	Mixed method	112 TB DOTS center/5 FGD (8–10 informants each group)	NA	Poor	Good	Poor	NA	Poor

**Table 7:** A summary of the studies included in this study.

First Author (year) Country, Period of Study	Sample Size/Type of HCWs	Transmission Control Measures			Results
		Administrative and Managerial	Engineering	Personal Protective	
Nazneen (2021). Bangladesh, Feb–Jun 2018 [34]	59 HCWS, 11 health settings/ 28 physicians, 11 nurses, 19 laboratory personnel, 1 project director	TBIC Guidelines: -None of the study respondents were aware of any written plan on TB IPC. -None of the workplaces had developed a workplace policy regarding TBIC. Committee/person in charge: -No IC coordinating body or person responsible for TBIC in the facilities. Training: -None of the respondents received training. Surveillance of HCW -No TB surveillance system for HCWs. Financial allocation -Almost all respondents mentioned that they did not receive any budget or instruction to conduct operational research. Triaging/separation of suspected or confirmed patients -Not consistent in triaging or isolating presumptive or TB patients and conducting counseling on cough etiquette. TB education -Inconsistent in delivering tuberculosis education to patients.	Ventilation -Good ventilation (natural ventilation). -Half of the facilities had functioning exhaust fans. UVGI -No UVGI device.	Availability of respirator -Staff at private and TB specialty units were only given N95 respirators. -Other staff were not provided. Usage of respirator -Laboratory personnel in TB specialty hospitals tended to wear N95 respirators and gloves to ensure TB IPC when closely handling TB specimens. Fit testing -None of the staff had fit testing or received any training on N95 respirators use.	-This study identified poor implementation of TBIC measures in health settings. -Limited knowledge of the guidelines, lack of hospital-level policies, unaware of HCWs toward TBIC policies, unavailable supply of N95 for tertiary care, and the health settings that prioritized patient’s management over TBIC resulted in poor implementation of the TBIC.
Engelbrecht (2018). South Africa, Oct–Nov 2015 [35]	41 PHC facilities/41 nurses	TBIC guidelines: -30% had written the IC plan. Committee/person in charge: -63.4% had an IC committee. Training: -44% had attended TBIC training. Triaging/separation of suspected or confirmed patients: -63.4% reported the separation, but 26.8% observed had separate presumptive TB patients. Others: 73.2% reported that coughing patients were provided masks, but only three facilities had masks available for patients, while observation results showed only two facilities had coughing patients wearing masks.	Ventilation -Most facilities reported used open ventilation. -30.3% observed used open ventilation. UVGI -Not mentioned.	Availability of respirator -22% of facilities did not have disposable respirators in stock. Fit testing -22% of respondents had undergone fit testing.	-TBIC was poorly implemented with low compliance on facility control measures and environmental controls measures. -Self-reported good TBIC practices were high, but by observation, the findings were different.
Chapman (2017). The Dominic Republic, August 2014 [33]	9 HCWs/7 physicians, 2 nurses	Surveillance of HCW -No national active surveillance system for TB HCWs. Triaging/separation of suspected or confirmed patients -Absence of isolation units. Others -Low provider to patient ratio.	Not evaluated	Availability of respirator -Limited protective mask provided.	-Perceived barriers identified as i. sense of invincibility of HCW; ii. a personal belief of HCW related to direct patient communication; iii. low HCW to patient ratio; iv. absence of TB isolation units for warded patients, very limited availability of respirators.
Engelbrecht (2016). South Africa, Sept–Nov 2015 [36]	41 facilities, 236 HCWS/202 nurses, 34 community HCW	TBIC guidelines: -72.9% had good TBIC practices. Training: -57% had received training on TBIC. Triaging/separation of suspected or confirmed patients -No separation in suspected TB patients with others. TB education -Good level of knowledge among HCWs. Others -80.4% had positive attitudes toward TBIC practices. -32.9% of respondents did not provide a mask to coughing patients.	Ventilation -95.2% of facilities well-implemented environmental control- they opened window; however, the observation revealed only 29.3% engaged in the practice. UVGI -Not mentioned.	Availability of respirator -78% N95 respirators were available in 32 facilities. Usage of respirator -52.2% always wore an N95 respirator when collecting sputum from suspected TB patients. -15.4% never used an N95 respirator in the TB consultation room. -Observation revealed 12.2% of facilities having tuberculosis nurses wearing N95 respirators. Fit testing -Not evaluated.	-Positive attitudes and good levels of knowledge were the main factors associated with good TBIC practices. -Good TBIC practices were reported by 72.9% of the respondents; the observation revealed different results. -For every unit increase in attitudes, good practices increased by 1.09 times. -Respondents with a high level of knowledge were four times likely to have good practices.
Godfrey (2016). LMIC, Feb 2013–Dec 2014 [37]	33 NIAID funded clinical research site	TBIC guidelines: -81% performed and documented regular audits of their SOPs. -22% of sites had all the evaluated TBIC elements in place. Committee/person in charge: -60% had IC officers. Surveillance of HCW -61% of sites had HCWS annual screening. Triaging/separation of suspected or confirmed patients -71% of sites promptly identified and segregated TB patients. -93% had separate waiting areas.	Ventilation -81% had well-ventilated sputum collection areas. UVGI -Not evaluated.	Availability of respirator -PPE was present in 97% of the sites. Fit-testing -43% were fit-tested.	-Sites with TBIC officers were more likely to have TB standard operating procedures, including monitoring of the policies and performing regular surveillance of HCWs.
Kuyinu (2016). Nigeria, March–July 2014 [38]	20 facilities, 10 HCWs	TBIC guidelines: -None of the clinics had a TBIC plan. Committee/person in charge: -30% of facilities had a dedicated person/committee responsible for TBIC. Training: -10% of staff were trained on TBIC. TB education -95% of facilities provided education to patients on cough hygiene. Others -No clinic consistently screened patients for cough. -60% consistently provided masks to patients who were coughing, but on observation, only 20% of facilities consistently provided masks.	Ventilation -60% of the facilities had adequate ventilation. -All clinics used mixed ventilation (mechanical and natural). UVGI -None of the facilities had UVGI. Others 10% of the clinic had designated sputum collection areas.	Availability of respirator -20% of the facilities had N95 respirators available. Usage of respirator -95% of staff did not use N-95 respirators.	-TBIC implementation was poor in health facilities in Ikeja, Nigeria. -Weak managerial support, poor funding, lack of space and staff had been identified as barriers to the implementation of TBIC.
Malangu and Mngomezulu (2015). South Africa, Feb–March 2012 [39]	52 health facilities	TBIC guidelines: -67.3% had a written infection control plan. Committee/person in charge: -76.5% existence of an infection prevention and control committee. Training: -62% had evidence of training being conducted in the last 6 months. Surveillance of HCW -80% of facilities complied with training staff with TBIC and screening staff for TB. TB education -All facilities complied with the requirement of educating patients. Others -All but one facility complied with the requirement of keeping a register for TB suspects. -All facilities complied with providing (IPT) to HIV-infected staff.	Ventilation -Most facilities did not comply with ventilation measures. UVGI -Only 20% of facilities used UVGI in a high-risk area. Others -Only 23.6% of the facilities complied with the position of staff according to airflow.	Availability of respirator -80% of facilities complied with making the N95 mask available to staff.	-The compliance of implementation of TBIC was low, with 48.6% of the TBIC measures complied with by at least 80% of the facilities.
Mugomeri (2015). South Africa, January 2012 [40]	55 nurses	TBIC guidelines: -58% reported using guidelines at least once a week. -22% reported inaccessibility to the guideline (keeping the guideline by certain nurses). Committee/person in charge: -94.6% were aware of the availability of the IC Committee and the guideline. TB education -71% reported they educated patients about tuberculosis daily.	Not evaluated	Availability of respirator -PPE was inadequate. -Lack of at least 1 piece of equipment specified in TB control was reported by respondents. Usage of respirators -There were cases with allergies with the PPE reported.	-There is poor adherence to TBIC guidelines by nurses in Lesotho (43.6%). -Factors that were significantly associated with the nonadherence were fear of occupational tuberculosis, lack of equipment, inadequate staff, and inaccessibility to the guideline.
Naidoo (2012). South Africa, 2009–2010 [41]	51 PHC	TBIC guidelines: -22% had infection control policies. -12% had an occupational tuberculosis management policy. Committee/person in charge: -20% had an infection control committee. Training: -8% provided in-service IC training to HCWs. Triaging/separation of suspected or confirmed patients -26% had triaged patients with cough symptoms. -31% had dedicated nurses and dedicated isolation rooms. -20% had dedicated room for TB patients only.	Ventilation -All rooms relied on natural ventilation, but in most of the clinics, windows remained close for the entire day. -53% had ACH less or the same as 12. UVGI -Not mentioned.	Availability of respirator -22% had N95 masks available for staff use. Usage of respirator -29% HCWs received basic training on respiratory protection from senior nurses. -During observations: no nurse was observed to be using N95. Fit testing -No fit testing was conducted.	-Findings show generally poor infection control practices at these facilities. -Limited infection control practices exist in clinics with a high TB burden in Kwazulu-Natal, South Africa. -No difference in clinic with and without infection control committee.
Kanjee (2011). South Africa, July–Sept 2007 [42]	57 HCWs	TBIC guidelines: -No TB IC policy or monitoring was in place. Triaging/separation of suspected or confirmed patients -TB cases or suspects were not routinely identified or expedited through services. -No separation in the waiting area. TB education -77.4% reported that always informing patients about cough hygiene. -32% of admitted TB cases wore masks.	Ventilation -69.1% reported that doors and windows were always opened in their work area. -Direct observation during winter day differed: 35% of outpatient tuberculosis offices opened windows, while that of the radiology department was 99%.	Usage of respirator -43.6% claimed that they always check for a tight facial seal when using respirators. -54.7% reported that they always use a respirator when in a room with TB patients.	-Knowledge and attitudes were supportive of TBIC implementation. -More than 90% of respondents were able to recognize classic tuberculosis symptoms.
Malotle (2017). South Africa [43]	285 HCWs, 144 nurses, 15 doctors, 82 support staff, 10 laboratories, 33 administrative staff	TBIC guidelines: -37.2% were unaware of the guidelines. -62.8% of respondents were unaware of the hospital management protocol. Training: -Low training was provided to HCWs, with 42.8% of them reporting contact with TB patients received TBIC training, 20 % received training on PPE in general, and 25.1% received training on respirator usage.	Not evaluated	Availability of respirator -62.2% of the HCWs reported that N95 respirators were always or sometimes available. Usage of respirator -44.9% of HCWs reported ever using respirators when managing patients with confirmed TB or presumptive TB.	-Despite available policies and guidelines, the gaps in the training of HCWs on how to protect themselves remained problematic. -Lack of training is closely associated with lack of protection.
Tamir (2016) Northwest Ethiopia, Jun–Sept 2014 [44]	647 HCWs, 35 health centers. 53.1% nurses, 12.8% health officers, 11.1% laboratory technologist, 10.9% midwives, 12% pharmacy	TBIC guidelines: -72.9% of respondents were aware of the presence of the TBIC plan. -75.8% of the respondent were aware of the presence of national guidelines for TBIC. Training: -34.5% of study participants were trained on TBIC. Surveillance of HCW -71.6% conducted screening at their workplace. Triaging/separation of suspected or confirmed patients -28.4% reported that tuberculosis suspect was not routinely identified in their departments. -No separate waiting area for tuberculosis patients. TB education -60% of the participants reported that they always informed coughing patients about cough hygiene. -54.8% reported that they were giving health education to tuberculosis patients and suspects.	Ventilation -89.1% reported that they always leave doors and windows opened during healthcare service provision. -Environmental control was not periodically maintained. UVGI -No other alternative like UVGI.	Availability of respirator -12% reported that there was a respirator in their working facility. Usage of respirator -23.5% reported they use respirators in a room during healthcare provision of TB patients/suspects.	-TBIC was not implemented effectively. -Reasons for not practicing TBIC among HCWs were structural barriers, such as lack of space for separation, understaffing, lack of managerial supports, and lack of motivation with negative attitudes of the HCWs.
Nawa (2020). Namibia, 2016–2017 [45]	3 Namibian hospitals/171 HCWs	Surveillance of HCW -HCWs screened for TB in all hospitals and clinics. Triaging/separation of suspected or confirmed patients -No overcrowding was observed in waiting rooms and hallways in all 3 hospitals. TB education -All hospitals had well-displayed signs and posters on coughing etiquette.	Ventilation -Poor ventilation (natural and mechanical) was observed in one of the hospitals. UVGI -Lack of UVGI machines.	Availability of respirator -Observation found that N95 respirators were available for use by HCWs except for one hospital -54% of respondents said that N95 respirators are always available from other wards. Usage of respirator -The majority of respondents from OPD/casualty wards confirmed that they do see patients without N95 respirators. Fit testing -Majority had a record of trained and fit testing for staff.	-The overall compliance of HCWs toward TPMs was good. -Factors associated with the risk of HCWs contracting TB were continuous exposure to tuberculosis patients, being in contact with undiagnosed patients, poor ventilation at health facilities, not following infection control measures, and overcrowdedness at hospitals.
Mekebeb (2019). South Africa [46]	2 hospitals/2 occupational health nurses	TBIC guidelines: -There was a TBIC plan and committee in hospitals, but it was only documented on paper in the clinic. Triaging/separation of suspected or confirmed patients -Fast-tracking, making outdoor waiting areas were noted to be implemented inconsistently. TB education -Cough hygiene education was noted to be implemented inconsistently. Others -Providing masks to cover cough was noted to be implemented inconsistently during the post-intervention assessment.	Ventilation -All consultation wards and rooms had open doors and windows. However, this was not constantly practiced in cold weather and at night. -No technical person/maintenance to assess the function of roof ventilators.	Availability of respirator -95 masks were consistently available. Fit testing -Fit tests were conducted regularly.	-TBIC was not implemented effectively in the facilities. -Assessment after a set of interventions did not show significant improvements in TBIC. -Most of the anticipated improvements were dependent on the HCWs’ adherence to local TBIC policies.
Kuyinu (2019). Nigeria, Oct 2016–May 2017 [47]	112 TB - DOTS centers. 5 FGD. Each group comprised 8–10 participants	TBIC guidelines: -21.4% had documented TBIC plans. Committee/person in charge: -Majority of DOTs (58%) centers had a dedicated TBIC officer or committee. Training: -57% of DOTS centers had staff that had been trained on TBIC; however, during the FGD, most of the participants claimed that they were not given any training or information pertaining to TBIC. Triaging/separation of suspected or confirmed patients -67.9% reported that patients were screened for cough, but by observation, only 50% of patients were screened for cough as of the time. -91% reported that triaging patients with cough. -By observation, 63% of the centers had separate waiting areas for people suspected of having TB. TB education -93% reported providing health education on cough hygiene and etiquette. -63% had information education displayed.	Ventilation -21% of the centers had adequate air exchange rates. -Consultations were carried out in open spaces in all facilities -75% had designated consulting rooms for TB activities and a combined ventilation system. UVGI -No UVGI was present in all facilities.	Availability of respirator -Only 13.4% of the centers had N95 respirators available for staff use. -37% provided PPE for staff or patients. Usage of respirator -The USE of N95 respirators by staff was observed in only 10% of the centers.	-TBIC measures at study centers were inadequate. -HCWs’ perception of being at risk of contracting TB was reported to affect the way they relate to TB pts.

Note: IC—infection control; TBIC—tuberculosis infection control; PPE—personal protective equipment; HCWs—healthcare workers; PHC—public health clinic; TPMs—tuberculosis preventive measures; LMIC—low middle-income country; NIAID—National Institute of Allergy and Infectious Disease; IPT—isoniazid prophylaxis therapy; ACH—air changes per hour; FGD—focus group discussion.
